# CO_2_ in the spotlight

**DOI:** 10.7554/eLife.08086

**Published:** 2015-05-13

**Authors:** Luis R Hernandez-Miranda, Carmen Birchmeier

**Affiliations:** Max-Delbrück-Centrum für Molekulare Medizin in der Helmholtz-Gemeinschaft, Berlin, Germanyluis.hernandes@mdc-berlin.de; Max-Delbrück-Centrum für Molekulare Medizin in der Helmholtz-Gemeinschaft, Berlin, Germanycbirch@mdc-berlin.de

**Keywords:** hindbrain, respiration, electrophysiology, genetics, transcription factors, mouse

## Abstract

Optogenetic techniques have revealed that retrotrapezoid neurons are essential for sensitivity to carbon dioxide.

**Related research article** Ruffault PL, D'Autréaux F, Hayes JA, Nomaksteinsky M, Autran S, Fujiyama T, Hoshino M, Hägglund M, Kiehn O, Brunet JF, Fortin G, Goridis G. 2015. The retrotrapezoid nucleus neurons expressing *Atoh1* and *Phox2b* are essential for the respiratory response to CO_2_. *eLife* **4**:e07051. doi: 10.7554/eLife.07051

**Image** Atoh1 neurons (blue) are found in the retrotrapezoid nucleus on the ventral (bottom) side of the brainstem

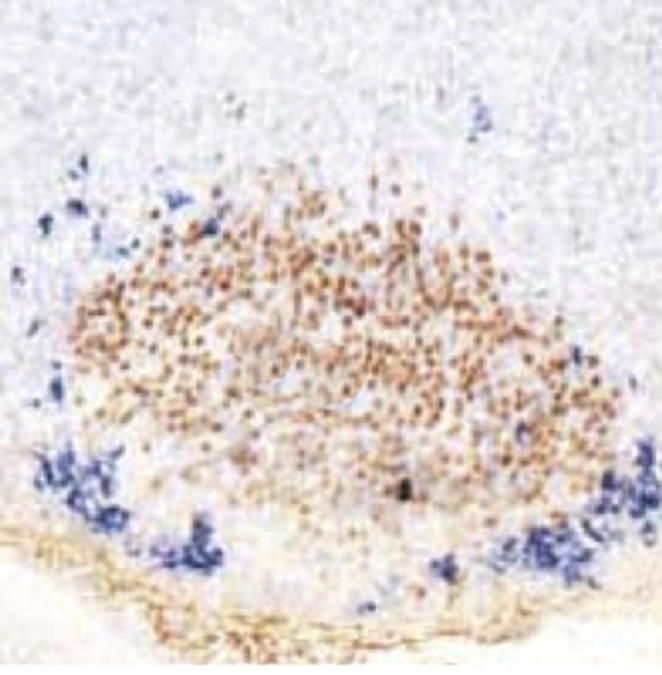

We breathe to get oxygen and to get rid of carbon dioxide (CO_2_). The precise monitoring of CO_2_ levels in the body is crucial because too much of it makes our blood acidic, which can have toxic effects. Under normal resting conditions, the concentration of CO_2_ controls our respiratory rate. For example, in most animals, including humans, high levels of CO_2_ lead to faster breathing. Moreover, having a weakened response to CO_2_ can be a life-threatening condition (Guyenet et al., 2010).

In spite of many years of research, the identity of the neurons involved in sensing CO_2_, and thus in controlling breathing, has been controversial (Guyenet et al., 2013). Now, in *eLife*, Jean-Francois Brunet of the Ecole Normale Supérieure and colleagues from France, Japan and Sweden report that neurons in the retrotrapezoid nucleus in the brainstem make up the CO_2_ sensor.

The retrotrapezoid nucleus is a tiny structure located in the ventral side of the brainstem—that is, it is towards the front of the brainstem in humans, but on the underside in mice and most other animals (Figure 1). These neurons rhythmically send signals to other neurons at normal body pH, and increase the rate at which they fire when the pH of the blood becomes acidic (Goridis and Brunet, 2010). The neurons in the retrotrapezoid nucleus are also directly connected to the preBötzinger complex (the structure in the brainstem that generates the breathing rhythm) and can adjust its activity. Mouse models in which the development of retrotrapezoid neurons is disrupted display slow breathing and have weakened, or blunted, responses to acidification (Sieber et al., 2007; Dubreuil et al., 2008, 2009; Goridis et al., 2010; Ramanantsoa et al., 2011). However, a recent study reported that retrotrapezoid neurons, particularly in newborn mice, might not be required for CO_2_ sensitivity (Huang et al., 2012). This cast doubts on the precise role of the retrotrapezoid nucleus.

**Figure 1.**
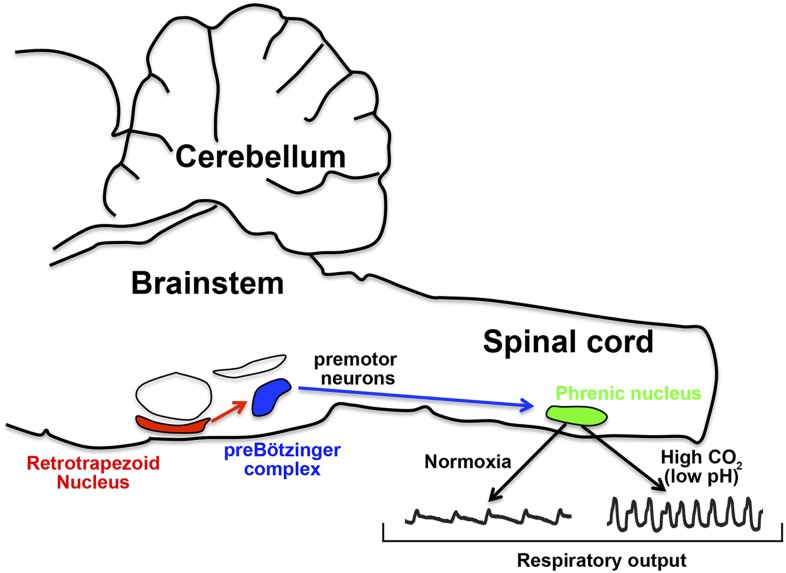
How the nervous system responds to CO_2_ in the blood. In mice, the retrotrapezoid nucleus (red) contains about 600 neurons and is directly connected to the preBötzinger complex (blue). Under normal levels of oxygen (normoxia), the activity of the preBötzinger complex controls the firing rate of neurons in the phrenic nucleus (green) by sending signals via premotor neurons. Phrenic neurons control the muscles involved in breathing and therefore control respiratory output. Ruffault et al. report that when levels of CO_2_ are high (which lowers blood pH), the retrotrapezoid neurons increase their firing rate: this adjusts the activity of preBötzinger complex and increases the firing rate of the phrenic neurons, which in turn increases respiratory output in order to eliminate more CO_2_.

Retrotrapezoid neurons send signals that activate, or excite, other neurons. Brunet and colleagues—who include Pierre-Louis Ruffault as first author—combined mouse genetics and optogenetic techniques to selectively stimulate specific retrotrapezoid neurons with light (Ruffault et al., 2015). This revealed that retrotrapezoid neurons adjust the activity of the phrenic nerve—the motor nerve that controls the muscles involved in breathing—by activating phrenic motor neurons via the preBötzinger complex (Figure 1). These results provide conclusive evidence of a direct association between the activity of retrotrapezoid neurons and respiratory motor neurons.

Retrotrapezoid neurons develop from progenitor cells located in the dorsal side of the brainstem (that is, towards the back of the brainstem in humans, but on the topside in mice). At first, these progenitor cells express a transcription factor called Phox2b. Later in their development, they start to co-express a transcription factor called Lbx1 and migrate towards the ventral brainstem (Sieber et al., 2007), where they switch on the expression of a third transcription factor called Atoh1. This means that these neurons are defined by the co-expression of these three transcription factors, and mutant mice that lack any of these three fail to develop a retrotrapezoid nucleus.

Ruffault et al. observed a blunted response to CO_2_ at birth in mutant mice that lack Atoh1, which is similar to the weakened response seen in Phox2b mutants (Dubreuil et al., 2008, 2009; Goridis et al., 2010; Ramanantsoa et al., 2011). In Atoh1 mutants, the neurons that are intended for the retrotrapezoid nucleus are born, but they fail to migrate to the ventral brainstem. And while these incorrectly positioned ‘retrotrapezoid’ neurons remain sensitive to pH, they cannot adjust the activity of the preBötzinger complex.

There are many thousand types of neurons in the brainstem, but only two populations of neurons are known to co-express Phox2b and Atoh1 (which Ruffault et al. call ‘Phox2b^on^/Atoh1^on^ neurons’). Only one of these populations is found in the retrotrapezoid nucleus, and the other population could be removed without any effect on CO_2_ sensitivity. Ruffault et al. then explored what happened when they silenced or activated the Phox2b^on^/Atoh1^on^ neurons within the retrotrapezoid nucleus. In newborn mice, this interfered with normal breathing and the response to pH, which illustrates that these neurons are indeed necessary to sense CO_2_ and adjust breathing accordingly at birth.

But, this is not the full story. Ruffault et al. found that the response to CO_2_ partially recovers in maturing animals that lack the retrotrapezoid neurons. This suggests that, later on in life, other regions of the brain are also involved in sensing CO_2_. However, when CO_2_ levels increase, animals that lack retrotrapezoid neurons respond by only mildly increasing their breathing rate, and the full response to CO_2_ is largely impaired (Ruffault et al., 2015).

Congenital breathing disorders in humans are rare but represent an immense burden to the affected individuals and their families (Ramanantsoa and Gallego, 2013). These diseases are life threatening because the afflicted patients breathe slowly, especially during sleep. In spite of their relevance and impact on public health, our current knowledge about these diseases is fragmented, and the only treatment for these conditions is mechanical ventilation of the patients. Mouse genetics allows us to explore the breathing centres in greater detail, and opens avenues to test new drug treatments that are designed to improve breathing. The work of Ruffault et al. represents a stringent study on the function of the retrotrapezoid nucleus, a breathing centre that is associated with congenital respiratory disease, and provides an animal model for future therapeutic research.
